# The Invisible Enemy and Our Methods of Defense*Annual Oration, Texas State Medical Association, delivered at the Opera House, San Antonio, Texas, April 27th, (ult.), by Dr. Matthew M. Smith, of Austin, Texas.

**Published:** 1899-05

**Authors:** 


### The Invisible Enemy and Our Methods of Defense.*

*Mr. President, Members of the Texas State Medical Asssociation, Ladies and Gentlemen:*

I appear before you this evening with a feeling of much diffidence, since I follow your honored and eloquent retiring president, whose beautiful and splendid address has reached the hearts of this entire audience. I recall the elegant reception which your Committee of Arrangements has prepared for our entertainment immediately after my remarks, and I shall therefore be brief.

I represent, as you know, upon this occasion, the representative body of the regular medical profession of the State, which numbers about 5000 physicians. I bring to you a message from these physicians, and ask for it your careful consideration, since their education and intimate knowledge of the causes of disease and its prevention makes their opinion in regard to the needs of medical legislation for the protection of the lives of the people of the State of the very greatest importance to you all.

My subject as announced by your chairman is: "The Invisible Enemy and Our Methods of Defense." The United States, as you know, has been at war with another great nation of the earth; a

---

*Annual Oration, Texas State Medical Association, delivered at the Opera House, San Antonio, Texas, April 27th, (ult.), by Dr. Matthew M. Smith, of Austin, Texas.

nation whose history three centuries ago, at the time of Philip II, would show it to have been the mightiest of the European powers. But its bright past has faded and vanished, and the last great blow to its downfall was when the United States, within a few short months, captured a large part of its army, destroyed its immense navy and claimed as a settlement its richest possessions. Since the Hispano-American war we have been engaged in battle with our latest territorial expansion acquirement, the "Spanish Philopena." Throughout these wars, whether on land or sea, the enemy was visible and life-size, and could be definitely located and attacked.

But we now have in existence and in constant operation all over this fair land an invisible war, a fearful war, one where the enemy is well concealed, and can not be seen by the untrained eye; yet is constantly in our midst, scattering death and destruction all around us. Not only does it attack in the open, but it charges from the rear, in ambush and in the dark hours of the night. Never recognizing any flag of truce, it doubles and charges when the victim is down, and shows no quarter. Waging eternal warfare at all times and in all seasons, attacking the rich and the poor, the white and the black and the high and the lowly. It is no respecter of education, wealth, color or previous condition of servitude. I refer to disease in all of its forms. Only a few years ago it appeared along our sea coast as yellow fever. In the neighborhood of Fort Worth and elsewhere recently as the deadly cerebro-spinal meningitis. In your neighboring city, Laredo, a few weeks ago as smallpox. Here and there throughout the State as diphtheria, pneumonia, typhoid fever and other forms of disease; but existing all over the country as consumption, with your fair city as a point of mobilization, because of your climatic conditions. Did you know that this fearful disease kills in its various forms at least one-seventh of the human family? Think for a moment of what an immense death rate, destroying the lives of more than 200 people every twenty-four hours in the United States alone. If one looks into any family history, consumption's sad story will be told in some of its forms, as truly expressed by Longfellow when he said:

"There is no flock, however watched and tended,
But one dead lamb is there.
There is no fireside, howso'er defended,
But has one vacant chair."

Disease, or the invisible enemy, not only killed more of the American soldiers in the late war with Spain and the Philippines than did the bullets of the enemy, but in the civil war, waged so fiercely for years, where the most brilliant land battles in the world's history were fought, still disease killed more of our gallant soldiers, both Federal and Confederate, than were slain in battle or died from wounds received therein. Thus, you see what a powerful enemy is disease.

Suppose we see what the State of Texas is doing in the way of protection against this powerful enemy, and in my remarks I do not wish to be understood as in any sense condemning what has been done, but I make this reference to show how much has been left undone which is of far greater importance to you all.

The present Legislature learned there existed a fatal disease to cattle in this State, and it appropriated money to employ an expert bacteriologist to study the disease, known as "black leg" in cattle, ascertaining if possible its cause and suggest a remedy for its eradication. Again, the same Legislature learned there existed a fatal disease to cotton; that during the past year certain epidemics of the disease occurred here and there over the State, and although Texas raises more cotton than any State in the Union, and the crop even sold as low as four cents per pound in 1898, still the present Legislature appropriating money to employ an expert entomologist to study this cotton disease, ascertain all about the microbe known as "bolus weevili," learn a method for its destruction, or, if necessary, quarantine it.

But what has been done for man? He is a stockman, a farmer, also a voter—yea, more, and has the lives of a wife and children to protect. He and his family have been dying rather frequently of late over the State. Not from one, but from many diseases. Not in cotton-growing season, but the year around. Not in certain localities, but all over the State. From cerebro-spinal meningitis, smallpox, diphtheria, typhoid fever, scarlet fever, consumption and many other forms of disease of a preventable nature. Yet the great State of Texas has made no special appropriations to employ expert bacteriologists and sanitarians to study the causes of the outbreaks of these diseases, suggest remedies for their prevention or final eradication from our midst. The only thing the lawmakers seem to think is necessary in the way of medical legislation is an appropriation for the quarantine system which guards the coast line against yellow fever, a disease no longer to be dreaded with the observance of suitable health laws and sanitary surroundings, as has been clearly demonstrated by the city of Memphis, Tenn., since the erection of its splendid system of sanitary sewerage; and which also makes an effort to isolate or quarantine smallpox, but which is a preventable disease, and would seldom be seen in the State if compulsory vaccination was enforced by law, which is really necessary in this State on account of the great extent of territory exposed to Mexico, where the disease constantly prevails.

Thus it is that a majority of the legislators, most of the people, largely the press and not a few doctors seem to think the public health is well protected and safe if quarantine is enforced along the coast line. Such a belief reminds me of the farmer who kept his sheep well housed and guarded for fear that a stray wolf from without might kill an occasional lamb, forgetting that the entire flock within was dying from starvation, lack of exercise and the dry rot. So with the State. It guards coast line and pickets the Mexican border to keep out two diseases, the one of seldom occurrence and the other clearly preventable, while neglecting the internal diseases which strike at the heart of the people and kill their thousands annually.

Dr. John S. Billings, in an address delivered before the American Academy of Political and Social Science at Philadelphia in 1891, struck the key-note to the situation when he said: "Every physician in active practice is familiar with the astonished, incredulous and slightly offended aspect of the man who comes to him for a prescription for a little dyspepsia, or hoarseness, or dizziness, or numbness, and is told that this is the beginning of the end, and that henceforth he can not follow his favorite habits and pursuits if he wishes to preserve his life. And this is true to a certain extent of communities as well as individuals. It is hard to persuade a city that it is ill or is in danger of becoming ill so long as the trade pulse beats strongly and clearly, and still harder to induce it to submit to any treatment which may slacken this pulse even temporarily."

This explains fully, you see, the people's apathy to public health matters. They rush through life thinking only of business, and forgetting entirely, or at least not contributing their individual part towards a public demand for suitable laws for the protection of their own lives and those of their families.

To the extent that a State reduces the amount of sickness among its people by the enactment of suitable public health laws, just to that extent does it reduce the amount of legitimate business among its physicians. Yet there are all over this State physicians (with no ax to grind) patriotic and philanthropic enough to loyally advocate and even spend their time and money endeavoring to have enacted suitable medical legislation for the prevention of disease. Is it not time, then, that the people throughout this State who, being most benefited, and with everything to gain and nothing to lose, should come to their assistance and demand at the State's hands the first great law of nature?

Did any one present here to-night ever try to learn from the insurance and statistics department at Austin how many bushels of potatoes were produced in the State for a year, how many bales of cotton were marketed, or how many pigs were reared? If not, I can assure you that such information was available formerly from the data collected by means of legislative enactment for that purpose; but on the other hand, if you endeavor to ascertain how many people were born within the State for a year, or the number that died for the same length of time, or the causes of death, you will find it impossible to obtain such information, because the State has never made provision for keeping such important data. The one was kept, I suppose, because it dealt with money values, the other was not because it dealt with life values. Or in other words, the great State of Texas keeps a system of vital statistics for its pigs, but none for its people.

There is no excuse for such a condition of affairs in Texas. Upon many different occasions the Texas State Medical Association has appointed committees to prepare and present before the Legislatures bills for the establishment of a State Board of Health and Vital Statistics. During the present Legislature such a bill was presented by a committee from this Association, through its chairman, the veteran sanitarian, Dr. R. H. Harrison, of Columbus, Texas, who not only labored earnestly in its behalf, but upon several occasions went to Austin, at a great sacrifice of time and at much expense, to urge its passage, but without success.

The great argument used against the bill was that it was an effort to do away with the State's quarantine system; which, however, was not contemplated or expressed in the bill. The objects of it being to establish a State Board of Health, whose duties should be not only to guard the State's health from outside diseases, but to also deal with all public health matters within the State, and have a board instead of one man to look after the entire health department of a State like Texas, with more land border than any State in the Union exposed to disease; with the second or third largest seacoast to guard and by all odds the largest amount of territory to be looked after, in fact, more than all the New England States, each of which possesses a State Board of Health composed of several members. In every other department of the State we find it requires more than one man to give efficient service, then in this, one of the most difficult and responsible branches of the government to fulfill, why not have others to assist the State Health Officer in guarding this large sea coast, immense land border and great extent of territory, where lives are sacrificed annually by the hundreds.

Texas guards the life and liberty of its citizens from visible enemies so carefully that when crime is charged it requires the unanimous opinion of the twelve jurors before whom the case is tried to convict or render the life sentence, and even then the higher courts and the governor's pardoning power can be resorted to. Yet, on the other hand, the State is so lax in its public health laws that it permits its citizens to be stricken down daily by diseases that would never have existed but for the State's unpardonable negligence. I suggest, if in order, that a bill be presented before the next Legislature, asking for permission to have the State tried for murder, because of its negligence in these public health matters.

But I have digressed somewhat, and I shall close after referring to a few of the most important methods of defense against disease. The best and surest safeguard against disease of all kinds is the individual's perfect health in every respect. Then a person born of healthy parents, properly fed and nourished, reared under strict hygienic rules and sanitary laws, given suitable exercise and recreation and free from vice or dissipation, in other words, complying with all of nature's health laws and violating none of them, such an individual will ordinarily be strong and healthy by nature, i. e., be sound in mind and member and free from ache or pain, and possess within himself a power of resistance that cannot be easily overcome by disease-producing organisms.

Another important method of defense against disease is a perfect public health system under the control of a State Board of Health, which would guard against outside diseases by enforcing, when necessary, suitable quarantine, but also investigating the causes of diseases all over the State and enforcing rules for its elimination, sending out to the public, when necessary, literature upon the prevailing disease and the best methods for their prevention; prohibiting the sale of impure and life-destroying drugs, looking after adulterated and unhealthy foods on the market, and taking cognizance of hundreds of other equally important matters that would naturally come before such a board.

Another great method of defense is for the State to require education and competency of its physicians and midwives. In the late wars, who did the United States place at the head of its army and navy, and who were given authority to direct and command the soldiers in times of great conflicts when they stood face to face with death, and where skill, education and practical experience meant so much towards the success of a battle? These high positions were only entrusted to men well trained in military knowledge and educated in the school of experience. Then another great defense against this invisible enemy, and one that is not only a duty, but also a necessity, is for the State to regulate the practice of medicine and midwifery, requiring those who have charge of the public health departments and who go forth to battle against disease to be highly educated and well experienced.

Certain requirements are exacted from a man before he is permitted to even conduct an ordinary pawn shop and speculate upon the financial misfortunes of others; yet but little more is required of its physicians, who practice medicine upon the bodily misfortunes of others. We have all over Texas, at present, people who profess to be able to diagnose diseases competently and treat them properly, who have never been inside of a medical college, seen a dissecting room, or read half a dozen medical books, yet they are given authority in the name of the State to go forth and treat the most complicated and intricate diseased conditions of its people. By law, such pretenders are placed on an equal footing with the highly educated, refined and conscientious physicians. Therefore, good sense would indicate, for your own protection, place in command to battle against disease in its multiform kinds, only physicians highly educated and suitably experienced, who have studied its habits, nature of invasion, methods of defense and power of resistance.

Again, our laws are such that a woman cannot teach any kind of a public school in the State, without first procuring by examination a certificate of qualification. Yet in every town and village, yea all over this State, there are women, both white and black, who profess to be midwives, without either education or qualifications, and entirely ignorant of the art which they profess to practice. These women are called in at the most critical period of a woman's life, at the birth of her child, a time, if the occasion should arise, when absolute cleanliness, thorough knowledge, cool judgment, skillful hands and rapid work not only means oftentimes the life of the child, but also the mother; and too often, I am sorry to say, neglect at this time is the beginning of a woman's ill health for life. And if the blind institutions of the State are consulted, they will show that one of the most common causes for the loss of man's greatest blessing, sight, is the neglect of the eyes for the first few days of life. There are many other methods of defense against this powerful enemy, disease, that I would like to discuss, but time will not permit me. I would have you understand, however, that what I said to you this evening is not the idle thoughts of a sanitary enthusiast; but on the contrary these conclusions have been reached after a careful study of this important subject, and I stand ready to prove and defend them at all times.

I have endeavored, in my crude way, to impress upon you the most important medical legislation needed in Texas, and I would have you know that the medical profession of this State is powerless to have such laws enacted without your assistance and co-operation. And in conclusion, ladies and gentlemen, you will pardon my reference to an often quoted subject, but it fittingly illustrates a thought I desire to leave with you. Sixty-three years ago, in this city, Wm. B. Travis, with 180 brave Texans, defended for days your Alamo against 6000 Mexican soldiers, who were invading your homes and devastating your country. And when this gallant commander realized death was inevitable if he resisted, he said: "We are determined to defend the Alamo to the last. I shall never surrender or retreat; victory or death." As you know, Travis and every one of his soldiers were slain in battle, fighting to the last for their country, their homes and their loved ones.

But change the scene to a few weeks later and what do we find. Gen. Sam Houston, at the head of his half-starved army, pushing his way by forced marches to the San Jacinto, where with 783 men he engaged in battle Gen. Santa Anna with more than twice the number, the flower of the Mexican army. And when Houston's men charged the enemy at double quick time, they raised the battle cry: "Remember the Alamo," and the recollection of the bloody massacre at San Antonio fired the hearts of those brave Texans and gave them Herculean strength and Roman courage, which caused them to rush into the thickest of the fight and win the battle, giving us the independence of Texas.

A similar condition exists in this State today. Texas is invaded by a powerful and invisible enemy, as I have described, which is devastating our country and destroying the lives of our people. The Texas State Medical Association and the physicians of the State, like the brave heroes of the Alamo, have been defeated and slaughtered on Austin's capitol hill, while fighting for laws to protect the lives and health of the people. The time is ripe for action, and our gallant commander, Dr. J. T. Wilson, is calling for volunteers to reinforce his army and press on to Austin in 1901 and again engage in battle the opponents of medical legislation, to fight for the passage of laws for the protection of the public health. And if the people at large will join his forces and then have turned on the enemy the powerful batteries of the public press of the State, which always stands ready to defend a just cause, and if this campaign is conducted in a true "Dewey-like style" until the next convention year, then even the politicians of the State will feel the people's pulse, shoulder their muskets, and declare publicly from the stump, "We are determined to defend the cause of public health to the last. Give my people needed medical legislation or give me political death."

Although our efforts in behalf of medical laws seem to have been in vain, still let us gain strength and courage for another fight from the remembrances of the battle of San Jacinto, so beautifully described by Thomas J. Rusk in his report to the President of Texas when he said: "The sun was sinking in the horizon as the battle commenced, but at the close of the conflict the sun of liberty and independence rose in Texas, never, it is hoped, to be obscured by the clouds of despotism."

